# Spectrally refined unbiased Monte Carlo estimate of the Earth’s global radiative cooling

**DOI:** 10.1073/pnas.2315492121

**Published:** 2024-01-22

**Authors:** Yaniss Nyffenegger-Péré, Raymond Armante, Mégane Bati, Stéphane Blanco, Jean-Louis Dufresne, Mouna El Hafi, Vincent Eymet, Vincent Forest, Richard Fournier, Jacques Gautrais, Raphaël Lebrun, Nicolas Mellado, Nada Mourtaday, Mathias Paulin

**Affiliations:** ^a^Laboratoire Plasma et Conversion d’Energie, Université de Toulouse, CNRS, Institut national polytechnique de Toulouse (INPT), Université Paul Sabatier (UPS), Toulouse 31062, France; ^b^Instituto de Astrofísica de Andalucía, Consejo Superior de Investigaciones Científicas (CSIC), Glorieta de la Astronomía s/n, Granada 18008, Spain; ^c^Laboratoire de Météorologie Dynamique-Institut Pierre Simon Laplace, Sorbonne Université, École normale supérieure (ENS), Université Paris Sciences et Lettres (PSL), École polytechnique, Institut Polytechnique de Paris, CNRS, Paris 75005, France; ^d^Institut de recherche en informatique de Toulouse, Université de Toulouse, CNRS, Toulouse 31062, France; ^e^Institut Mines-Télécom Mines Albi, Centre de Recherche d’Albi en génie des Procédés des Solides Divisés de l’Energie et de l’Environnement, CNRS, Campus Jarlard, Albi 81013, France; ^f^Méso-Star, Toulouse 31200, France; ^g^Centre de Recherches sur la Cognition Animale, Centre de Biologie Intégrative, Université de Toulouse, CNRS, Toulouse 31062, France

**Keywords:** climate change, radiative forcing, line-by-line, Monte Carlo

## Abstract

The Earth’s radiative cooling is a key driver of climate. Determining how it is affected by greenhouse gas concentration is a core question in climate-change sciences. Due to the complexity of radiative transfer processes, current practices to estimate this cooling require the development and use of a suite of radiative transfer models whose accuracy diminishes as we move from local, instantaneous estimates to global estimates over the whole globe and over long periods of time (decades). Here, we show that recent advances in nonlinear Monte Carlo methods allow a paradigm shift: a completely unbiased estimate of the Earth’s infrared cooling to space can be produced using a single model, integrating the most refined spectroscopic models of molecular gas energy transitions over a global scale and over years, all at a very low computational cost (a few seconds).

The Earth radiative budget plays a fundamental role in the climate system. Its global value drives the global mean surface temperature changes, its latitudinal variation drives the atmosphere and ocean circulation, its value in the atmosphere drives the mean precipitation amount, etc. (1). Building an estimate of it requires the very demanding task of dealing jointly with linear transport physics and molecular spectroscopy, while handling millions of rotation/vibration lines of greenhouse gases inside a heterogeneous multiple scattering atmosphere containing clouds. One of the most widely publicized diagnostic is the instantaneous forcing of greenhouse gases (${\text{H}}_{2}\text{O}$, ${\text{CO}}_{2}$, ${\text{CH}}_{4}$, etc.), defined as the sensitivity of the radiative flux at the top of the atmosphere or at the tropopause to a change in atmospheric greenhouse gas concentration, all other variables being kept constant. An accurate estimate of this sensitivity requires the same detailed physical modeling as for remote sensing, i.e., to include all the statistical physics and quantum mechanics responsible for the intensity and frequency shape of all the spectral lines. Estimates for climate studies require integration over a climatic period $[t_{1}$,$t_{2}]$, over the whole globe, over all frequencies ν, and over the whole height of the atmosphere (Fig. 1).

**Fig. 1.**
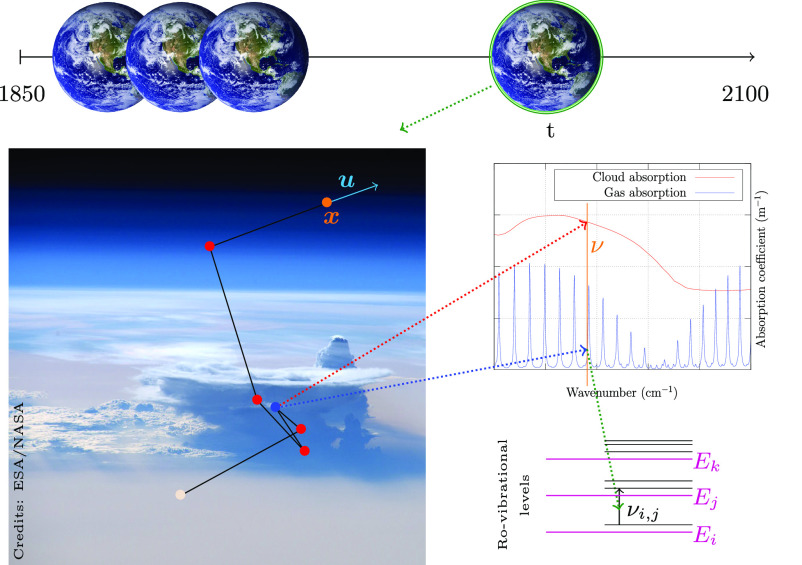
Complexity of the integration domain. The flux density at top of atmosphere (TOA), at a given location and a given time, is a double integral: over all frequencies and over the space of multiple-scattering paths. This flux density is integrated over all TOA locations and over a climatic period. The local thermodynamic state of the atmosphere (temperature T, total pressure P, partial pressure $P_{i}$ for species i) is different at each time during this climatic period, which is essentially translated into a time-varying line spectrum, associated with molecular states’ transitions broadened by frequency shifts and collisions, with Doppler line shapes in the upper atmosphere (no collision) that are very significantly sharper than Lorentz shapes close to the surface where pressure-broadening dominates.

The long-term average of top-of-atmosphere flux emitted by the atmosphere toward space, assuming local thermodynamic equilibrium, is given by integrals in space, time, and electromagnetic frequency:

[1] $$E_{LW}=\int_{t_{1}}^{t_{2}}dt\int_{0}^{+\infty }d\nu \int_{Globe}dx\int_{0}^{Z_{T}}dz\int_{\Gamma (x)}D\gamma 4\pi k_{a,\nu }(x,z,t)I_{\nu }^{eq}(T(x,z,t))T(\gamma ),$$

where x is the longitudinal/latitudinal location, z the altitude, and Γ the space of all sample paths γ starting at this location. $4\pi k_{a,\nu }I_{\nu }^{\mathrm{eq}}$ is the local atmospheric emission, where $k_{a,\nu }$ is the monochromatic absorption coefficient and $I_{\nu }^{\mathrm{eq}}$ the Planck function at the local temperature. T is either 1 if the path reaches the top of the atmosphere at altitude $Z_{T}$ and contributes to the top of atmosphere flux, or 0 if the path ends with absorption either by the atmosphere or the ground. The notation Dγ is used for the probabilistic measure of the multiple scattering, multiple reflection process describing the way photons interact with both the atmosphere and the ground. For a nonreflecting ground and a nonscattering stratified atmosphere, the statistics could be reduced to those of the azimuth angle cosine μ on [−1,1] and the path length l on [0,+∞[. For example, arbitrarily extending $k_{a,\nu }$ below the ground and above the atmosphere to represent complete absorption when reaching ground or space, Dγ would typically be written as:

[2] $$\begin{array}{c} D\gamma =\frac{1}{2}k_{a,\nu }\left(x,z,t\right)\exp\left(-\int_{0}^{l}k_{a,\nu }\left(x,z+\mu l^{\prime },t\right)dl^{\prime }\right)d\mu dl. \end{array}$$

The total absorption coefficient:

[3] $$\begin{array}{c} k_{a,\nu }\left(x,z,t\right)=\sum_{i=1}^{N_{t}}h_{a,\nu ,i}\left(x,z,t\right), \end{array}$$

is the sum of absorption due to many spectral lines, $h_{a,\nu ,i}$, each of which corresponds to a given quantum mechanical transition provided by spectroscopic databases such as Gestion et Etude des Informations Spectroscopiques Atmosphériques (GEISA) (2) or high-resolution transmission molecular absorption database (HITRAN) (3).

Computing the multiple integrals and the transition sums in Eqs. **1**–**3** with a standard discretization approach is a routine practice but computation times lengthen significantly when summing over many lines at each frequency. Alternative strategies have therefore been adopted by the community by reducing climate to a limited number of representative atmospheric profiles and/or by simplifying the spectroscopic description of gas absorption (1, 4, 5).

We report here that nonlinear Monte Carlo strategies now offer a practical way of bypassing these approximations and working directly with the most refined radiative transfer models, without sacrificing any spectral, spatial, or temporal dimensions. We even show that this Monte Carlo integration is fully insensitive to each of these integration dimensions and that to estimate the global mean radiative flux at the top of the atmosphere does not require more than a few seconds on a standard laptop (Fig. 2). This is a breakthrough when compared to conventional approaches to calculating radiative forcing and opens up research avenues in atmospheric radiative heat transfer.

**Fig. 2.**
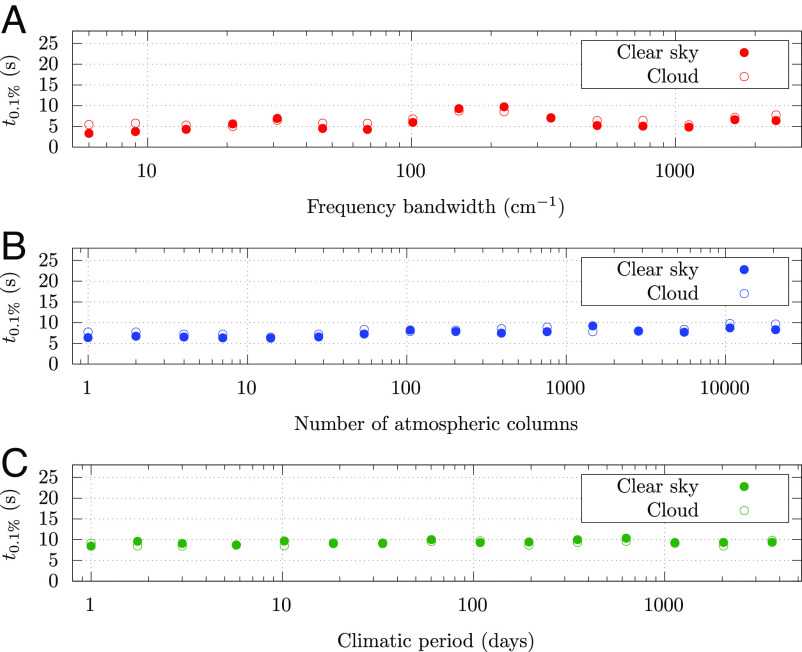
No sensitivity of computation time to the extension of the integration domains (∙ without and ° with scattering by 1D clouds). Time to perform a Monte Carlo computation of the radiative flux at the top of the atmosphere, with an uncertainty of 0.1%, on a personal computer with 12 CPUs, as a function of the size of the main integration domains: (*A*) a single time and a single column but different spectral integration ranges; (*B*) a single time, all infrared, but different spatial integration ranges (number of columns); (*C*) all infrared, the whole Earth, but different climatic period integration ranges. The sky is either clear or with an idealized high level cloud to show the very limited impact of including some scattering. Note that while the variance of the estimates is unaffected by the widening of the integration domains, the estimates of radiative flux are themselves affected. Only the last green dot represents the computation time required for a climatically meaningful quantity: the mean radiative flux over all thermal infrared domain, all the globe and a 10-y period.

Why haven’t such Monte Carlo simulations been performed before? The reason lies essentially in the absorption coefficient $k_{a,\nu }$ appearing inside the exponential of Beer’s extinction law in Eq. **2**. If $k_{a,\nu }$ were known and did not vary along the vertical axis, Monte Carlo integration in time, space, and frequency Eq. **1** would present no particular difficulty. However, $k_{a,\nu }$ is actually a computationally expensive sum over molecular state transitions that indeed vary sharply on the vertical axis, because of absorbing gas concentrations (${\text{O}}_{3}$, ${\text{H}}_{2}\text{O}$, …), pressure, and temperature. So the very computation of $k_{a,\nu }$ needs to be nested into this first-level integral through a nonlinear function (integrating the exponential of an integral). Unfortunately, when it comes to nonlinear combinations of integration spaces, Monte Carlo methods are notoriously inefficient, if not impossible to use (6, 7).

Breakthroughs have been reported in refs. 8 and 9. Here, we essentially retain the null-collision concept as a way to bypass the nonlinearity of the exponential function (10, 11). In short, the idea is to add virtual colliders that do not modify the solution of the equations such that their addition allows the true absorption coefficients $k_{a,\nu }$, which vary with location, to be replaced by a uniform collision coefficient, whose value is set to ${\hat{k}}_{a,\nu }$, the upper bound of the true ones. This virtual value is then used to sample the distance covered before the next collision. This distance is inevitably underestimated, and a rejection technique is used to ignore the virtual colliders. In such cases, the algorithm continues, sampling a new path length and a new probability of being absorbed, and so on until absorption.

In practice, once time, frequency, position on the globe and direction of propagation (t, ν, x, z in Eq. **1** and μ in Eq. **2**) have been sampled, we sample a path-length as if the photon were traveling through this upper-bound field ${\hat{k}}_{a,\nu }$ and move it to the collision location. At this stage, a standard null-collision algorithm would require defining a collision-rejection probability $P_{n}=\left({\hat{k}}_{a,\nu }-k_{a,\nu }\right)/{\hat{k}}_{a,\nu }$, where $k_{a,\nu }$ is a function of the local thermodynamic state of the atmosphere at this location. However, the calculation of $k_{a,\nu }$ represents by itself a very large computational cost due to the large number $N_{t}$ of molecular transitions that need to be taken into account in Eq. **3**. This is often replaced in line-by-line models by a costly pre-computation of look-up tables that are used to interpolate the value of $k_{a,\nu }$ at each location. In order to avoid this pre-computation step and obtain direct access to the spectroscopic data, we push the framework further to include the calculation of this sum in the Monte Carlo integration itself: In ref. 12, it was shown that when $P_{n}$ is an expectation, the corresponding random variable can be sampled and null collisions can be decided from the sample without ever estimating $P_{n}$. Here, the corresponding idea is to sample one transition and decide null collisions using this single transition. In this way, we have designed a fully unbiased spectrally refined Monte Carlo estimate: by enriching the path-space structure with orthogonal random visiting of energy transitions (*Material and Methods*). We also made use of machine learning techniques to further accelerate the computation of both ${\hat{k}}_{a,\nu }$ and the sampling of transitions. Here, the machine learning induces no uncontrolled source of uncertainty: These accelerations have been constructed in a strictly consistent way to ensure that the Monte Carlo estimate remains rigorously unbiased (*SI Appendix*).

We validated the fluxes against the state-of-the-art 4A-Flux line-by-line model (13) for a set of standard atmospheric columns. Using the 100 atmospheric profiles proposed by Pincus et al. (5), we obtained a radiative forcing of $2.73\pm 0.06\;W\;m^{-2}$ for a doubling of the ${\text{CO}}_{2}$ concentration by doing the difference between two mean fluxes, consistent with their mean estimate of $2.71\;W\;m^{-2}$. We then went one step further by performing a computation over the whole globe and over climatic periods. To achieve this, the atmospheric columns were first randomly sampled from three hourly outputs of a 10-y simulation of the Institut Pierre-Simon Laplace (IPSL) climate model (14) before the radiative calculation was performed. We obtained a global average clear-sky radiative forcing of $2.56\pm 0.06\;W\;m^{-2}$.

The calculations are very fast as it takes only a few seconds on a standard personal computer to estimate this flux, with a statistical error of 0.1%, obtained by launching about $N=10^{7}$ samples. As expected for a converging Monte Carlo code, the statistical error decreases with the square root of N and the computation time cost lies in the integration including the largest source of variance and does not increase when extending the size of the other integration domains (Fig. 2). For a given atmospheric column and a given relative uncertainty, the computation time required to estimate the radiative flux does not increase as the spectral range of integration varies from 6 cm^−1^ to the entire spectral range (Fig. 2*A*). Likewise, for a given spectral domain and a given relative uncertainty, the computation time does not increase when increasing the spatial (Fig. 2*B*) and the time (Fig. 2*C*) integration domains (*SI Appendix*).

We have thus devised a line-by-line Monte Carlo integration of TOA radiative flux at the scale of the Earth and for climatic periods, which is insensitive to the widening of frequency, spatial, and temporal integration domains, and which is valid for a scattering or nonscattering medium. Freeing the climate science community from the need for pre-computed approximation schemes, these estimates are now available in a matter of seconds on a desktop computer, thus opening up a wide range of perspectives in climate science (*SI Appendix*).

## Materials and Methods

Starting from the radiative transfer equation (with pure absorption for the sake of simplicity),

$$\begin{array}{c} u.\nabla I=-kI+kI^{\mathrm{eq}}, \end{array}$$

where I≡I(x,z,u) is the specific intensity in direction u, the simplest viewpoint on null-collision (11) is to consider virtual colliders as pure forward scatterers:

$$\begin{array}{c} u.\nabla I=-\hat{k}I+\hat{k}\left[\left(1-\omega \right)I^{\mathrm{eq}}+\omega \int_{4\pi }\delta \left(u-u^{\prime }\right)I^{\prime }du\right] \end{array}$$

with $I^{\prime }\equiv I\left(x,z,u^{\prime }\right)$ and single scattering albedo $\omega =\frac{\hat{k}-k}{\hat{k}}$, the Kronecker symbol δ representing the pure forward scattering phase function. Looking at each transition i as if it were an independent species, with $k=\sum h_{i}$, virtual colliders can be added to each transition, with ${\hat{h}}_{i}>h_{i}$ to write:

$$\begin{array}{c} u.\nabla I=-\hat{k}I+\hat{k}\sum P_{i}\left[\left(1-\omega_{i}\right)I^{\mathrm{eq}}+\omega_{i}\int_{4\pi }\delta \left(u-u^{\prime }\right)I^{\prime }du\right] \end{array}$$

with $\hat{k}=\sum {\hat{h}}_{i}$, $P_{i}=\frac{{\hat{h}}_{i}}{\hat{k}}$, and $\omega_{i}=\frac{{\hat{h}}_{i}-h_{i}}{{\hat{h}}_{i}}$. As for standard Monte Carlo algorithms involving mixtures, $\hat{k}$ is used to find the next collision location, $P_{i}$ to decide which transition was responsible for the collision, and $\omega_{i}$ to decide whether the collision is an absorption or a scattering event (here a rejection).

## Supplementary Material

Appendix 01 (PDF)

## Data Availability

Code data have been deposited in RadForcE (https://gitlab.com/yanissnp/RadForcE) (15).
