# Supplementary material for: Spectrally refined unbiased Monte Carlo estimate of the Earth’s global radiative cooling
Source: Proc Natl Acad Sci U S A. 2024 Jan 22;121(5):e2315492121. doi: 10.1073/pnas.2315492121 (PMC10835068; doi:10.1073/pnas.2315492121)
Supplement: Supplementary file 1 — Appendix 01 (PDF) [file pnas.2315492121.sapp.pdf]

Supporting Information for

Spectrally refined unbiased Monte Carlo estimate of the Earth's global radiative cooling

Yaniss Nyffenegger-Péré, Raymond Armante, Mégane Bati, Stéphane Blanco, Jean-Louis Dufresne, et al.

Yaniss Nyffenegger-Péré.
E-mail:nyffenegger@laplace.univ-tlse.fr

This PDF file includes:

Supporting text
SI References

Supporting Information Text

Extended Materials and Methods

Accelerated sampling. To accelerate the sampling of collisions and the associated transition, for each molecular species, a binary probability tree is built for a given frequency domain and for a set of thermodynamic conditions. For each tree, the spectral lines are first partitioned according to their central frequencies. In this way, the total spectral domain is split into narrow bands at the bottom level of the tree (one single line per leaf). In each narrow band, an approximate upper bound of the absorption coefficient is built based on its exact value at discretized frequencies. The choice of these frequencies is driven by the deterministic line-by-line approach (1). Finally, an upper level is built by merging pairs of neighbouring leaves, each upper node with its associated $\hat{k}$; and this is accomplished, level by level, up to the root node of the tree. For a given frequency, the upper-bound absorption coefficient is then readily obtained by querying data stored at the root level. At the same frequency, sampling a spectral line is quickly performed by descending the tree (2), sampling a node at each level (using the data stored at this level) down to a leaf, therefore deciding a line, meanwhile constructing P_i as the product of the successive node-sampling probabilities. With regard to variations in thermodynamic conditions, the main difficulty lies in the pressure, as it modifies the width of the spectral lines and varies by several orders of magnitude. Several trees were built at specific pressure conditions and the required variables were then linearly interpolated to local pressure. The complexity of the precomputation algorithm is $\Theta(n)$, it linearly depends on the number of transitions n. For instance, considering all spectral lines of CO_2 over the infrared thermal range (≈ 255000 transitions) and over 8 different pressures, the CO_2 binary tree takes up to 4GB of memory space for a computation time of about 1 minute (on a i7-1370P laptop processor, using a single execution thread). A change either in pressure condition or in the spectroscopic database requires rebuilding the tree. A new molecular species can be added independently by building its own tree.

As highlighted in the main text, the only impact of the way the sampling procedure is tuned is on convergence speed. This is nothing but an importance sampling approach: the estimate is unbiased.

Statistical convergence. The fact that there is no significant sensitivity of computation time to the number of columns reflects the greater complexity of frequency integration compared with that of thermodynamic states (in space and time). Even a spectral band of width 6cm^{-1} in a single column (such as that of the first red point in Fig.2) includes a large number of spectral lines and requires 10^7 samples for a 0.1% convergence. This requires in turn that the sample-size for columns is also 10^7. The fact that we still get the same 0.1% convergence whether we always draw thermodynamic state from the same column or draw from the large number of columns describing the global climate on a climatic period (up to 6×10^8 columns in the examples here) means that integration over thermodynamic states is here a significantly better-behaved problem than spectral integration.

Possible future avenues of research. New areas of research seem to be conceivable thanks to the work presented here, and some are already being explored. These include the following:

- The possibility of including the 3D radiative effects of aerosols and clouds (3, 4), provided we know their complete geometry, would make it possible to have a complete reference model which, as far as we know, does not yet exist.
- Ray tracing makes it possible to produce original diagnoses that allow a renewed analysis of radiation exchanges: emission altitude, nature of the emitter, etc.
- In addition to the computation of the radiative flux, its sensitivity (in the form of partial derivatives) to some parameter perturbation can be estimated with minor additional computer cost (5–7).
- Random sampling can be an economical way of performing calculations or analyses on very large volumes of data, such as those from model outputs or satellite measurements. The ten millions atmospheric columns used here to calculate a flux with an uncertainty of 0.1% represent just 1.5% of the 10 years of data, every 3 hours, for a horizontal grid of $1.5^\circ \times 2.5^\circ$, a fraction that is considerably reduced when using data from models or instruments with very high spatial resolution.
- For planets other than Earth, the absence of inter-median calculations (such as the pre-calculation of spectral absorption coefficients) should enable a wide range of atmospheres to be explored more rapidly.

References

1. M Kuntz, M Höpfner, Efficient line-by-line calculation of absorption coefficients. *J. Quant. Spectrosc. Radiat. Transfer* **63**, 97–114 (1999).
2. MD McCool, PK Harwood, Probability trees in *Graphics Interface*. Vol. 97, pp. 37–46 (1997).
3. N Villefranque, et al., A path-tracing Monte Carlo library for 3-D radiative transfer in highly resolved cloudy atmospheres. *J. Adv. Model. Earth Syst.* **11**, 2449–2473 (2019).
4. R Lebrun, JL Dufresne, N Villefranque, A consistent representation of cloud overlap and cloud subgrid vertical heterogeneity. *J. Adv. Model. Earth Syst.* **15**, e2022MS003592 (2023).
5. GA Mikhailov, Monte-Carlo calculation of derivatives of functionals from the solution of the transfer equation according to the parameters of the system. *USSR Comput. Math. Math. Phys.* **7**, 274–281 (1967).

6. A de Lataillade, et al., Monte Carlo method and sensitivity estimations. *J. Quant. Spectrosc. Radiat. Transfer* **75**, 529–538 (2002).
7. M Roger, S Blanco, M El Hafi, R Fournier, Monte Carlo estimates of domain-deformation sensitivities. *Phys. Rev. Lett.* **95**, 180601 (2005).
